# Evaluation of the remineralization capacity of CPP-ACP containing fluoride varnish by different quantitative methods

**DOI:** 10.1590/1678-775720150583

**Published:** 2016

**Authors:** Selcuk SAVAS, Fevzi KAVRÌK, Ebru KUCUKYÌLMAZ

**Affiliations:** Department of Pediatric Dentistry, Faculty of Dentistry, Izmir Katip Celebi University, Izmir, Turkey.

**Keywords:** Hardness, Dental enamel, Tooth remineralization

## Abstract

**Objective:**

The aim of this study was to evaluate the efficacy of CPP-ACP containing fluoride varnish for remineralizing white spot lesions (WSLs) with four different quantitative methods.

**Material and Methods:**

Four windows (3x3 mm) were created on the enamel surfaces of bovine incisor teeth. A control window was covered with nail varnish, and WSLs were created on the other windows (after demineralization, first week and fourth week) in acidified gel system. The test material (MI Varnish) was applied on the demineralized areas, and the treated enamel samples were stored in artificial saliva. At the fourth week, the enamel surfaces were tested by surface microhardness (SMH), quantitative light-induced fluorescence-digital (QLF-D), energy-dispersive spectroscopy (EDS) and laser fluorescence (LF pen). The data were statistically analyzed (α=0.05).

**Results:**

While the LF pen measurements showed significant differences at baseline, after demineralization, and after the one-week remineralization period (p<0.05), the difference between the 1- and 4-week was not significant (p>0.05). With regards to the SMH and QLF-D analyses, statistically significant differences were found among all the phases (p<0.05). After the 1- and 4-week treatment periods, the calcium (Ca) and phosphate (P) concentrations and Ca/P ratio were higher compared to those of the demineralization surfaces (p<0.05).

**Conclusion:**

CPP-ACP containing fluoride varnish provides remineralization of WSLs after a single application and seems suitable for clinical use.

## INTRODUCTION

The first clinical sign of dental caries is the appearance of a white spot lesion (WSL) on the tooth surface that can be considered the initial stage of enamel demineralization^12^ ^,^ ^19^. If the demineralization process continues, the initial lesion may progress to cavity formation. Thus, enhancing remineralization of early carious lesions is an effective, non-invasive treatment for maintaining healthy dentition^19^ ^,^ ^20^ ^,^ ^26^.

Among the available strategies, the use of fluorides has been shown to be highly effective in managing WSLs^10^ ^,^ ^18^ ^,^ ^22^. Fluoride increases the remineralization of the outer enamel and decreases the demineralization of the inner enamel, resulting in significant mineral gain. However, some authors have suggested that using high concentrations of fluoride causes remineralization especially in the superficial part of the WSLs. This superficial layer might retard calcium and phosphate, inhibiting deeper remineralization and limiting the cosmetic improvement of the WSLs^10^ ^,^ ^25^. Therefore, there is a need to investigate other anti-carious agents that could have a synergistic effect with fluoride or be an alternative to enhance remineralization of dental caries.

Casein phosphopeptide amorphous calcium phosphate (CPP-ACP) is a technology based on amorphous calcium phosphate (ACP) stabilized by casein phosphopeptides (CPP)^7^ ^,^ ^8^. The integrity of the dental hard tissues relies on the oral fluids being saturated or supersaturated with tooth minerals^4^ ^,^ ^21^. The beneficial effect obtained from CPP-ACP is associated with the ability to localize calcium and phosphate in dental plaque in the proximity of the tooth, thus making it available when needed^4^ ^,^ ^21^. In recent studies, the remineralization potential of CPP-ACP combined with fluoride has been investigated, and a synergistic effect when they are administered together was found^4^ ^,^ ^7^ ^,^ ^10^ ^,^ ^21^ ^,^ ^24^ ^,^ ^27^. The remineralization capacity of CPP-ACP products has been studied in detail; however, to our knowledge, there is only one study that evaluated the ion release from fluoride varnish containing CPP-ACP, and no study was found about its efficacy on the remineralization of WSLs. Thus, there is a need to investigate the remineralization potential of this material.

Currently, preventive and minimally invasive dentistry are offering a wide variety of methods to detect and care for even the tiniest changes in hard tooth tissue. Selecting an appropriate treatment to remineralize WSLs and accurately and reliably monitor lesion progress or regression is the key to avoid the need for invasive treatments. Therefore, the purpose of this study was to determine the potential of CPP-ACP containing fluoride varnish to remineralize WSLs using four types of analysis: enamel surface microhardness (SMH), quantitative light-induced fluorescence-digital (QLF-D), energy-dispersive spectroscopy (EDS), and laser fluorescence (LF) pen.

## MATERIAL AND METHODS

The present study was approved by the Medical Ethics Committee under report number 2015/150.

### Sample preparation

Thirty bovine incisors free from clinically visible abnormality were used. They were cleaned of debris/stains with pumice, stored in a 1% thymol solution at 4°C, and used within 1 month of their extraction. The teeth were thoroughly rinsed and examined under a stereomicroscope (Olympus SZ61; Olympus Optical Co.; Tokyo, Japan). Any tooth with defects, erosions, or microcracks in its enamel surfaces was excluded. The soft tissue and calculus were removed mechanically from tooth surfaces with a scaler (U15/30 Hu-Friedy, Hu-Friedy Manufacturing Inc., Chicago, IL, USA). The roots were removed by sectioning approximately 2 mm below the cementoenamel junction and perpendicular to the long axis, using a water-cooled diamond disk (Isomet, Buehler Inc.; Lake Bluff, IL, USA). Then, the coronal part of each tooth was horizontally embedded in self-cured acrylic resin (Vertex Dental; Zeist, Netherlands) in prefabricated cylindrical molds, keeping the buccal surface exposed, and cured overnight. The exposed enamel surface of each tooth was polished with 600-, 800-, and 1000-grit silicon carbide paper for 5 s each to obtain a flat and smooth surface. Each specimen was coated with a clear nail varnish, leaving four enamel windows with approximately 3×3 mm exposed in the center.

### Preparation of the artificial caries lesion

Following preparation of the specimens, a control window was coated with acid-resistant varnish and artificial carious lesions were created on other exposed windows by demineralizing the specimens for 7 days in an acidified gel system. The gel was prepared by adding 100 mmol/L sodium hydroxide to 100 mmol/L lactic acid to give a final pH value of 4.5^1^. To this solution, 6% w/v hydroxyethyl cellulose was added while vigorously stirring. The final consistency of gel had a viscosity in the region of 100 cP. After the 7-day demineralization period, each specimen was cleaned of the demineralization solution with de-ionized water and stored at room temperature.

### Remineralization

At the end of the demineralization phase on each specimen, a demineralization window was painted with nail varnish. The other two windows were treated with the tested material (MI Varnish; GC Corp.; Tokyo, Japan). The varnish was applied in a thin, uniform layer on the surface with a disposable brush and remained undisturbed on the teeth for 4 h, according to the manufacturer’s instructions. Immediately after this application, each sample was placed in an individual container containing 10 mL of artificial saliva [MgCl_2_6H_2_O (0.148 mmol/L), K_2_HPO_4_ (4.59 mmol/L), KH_2_PO_4_ (2.38 mmol/L), KCl (8.39 mmol/L), calcium lactate (1.76 mmol/L), fluoride (0.05 ppm), sodium carboxymethylcellulose (2.25 mmol/L), methyl-4-hydroxybenzoate (13.14 mmol/L); pH 7.2]. All samples were placed in an incubator at 37°C with 5% CO_2_ for 1 week and 4 weeks, and the enamel of the two windows was remineralized_._ At the end of the 1-week remineralization phase, one exposed window was painted with nail varnish. During the 4-week remineralization procedure, the artificial saliva was renewed daily. Following the 4-week test period, the nail varnish was removed from all the specimens with acetone. Then, the baseline, demineralization, 1-week post-treatment, and 4-week post-treatment specimens were tested by the following four methods: SMH, QLF-D, EDS, and LF pen analyses.

### QLF-D analysis

The mean fluorescence loss for each enamel window was assessed with QLF-D Biluminator™ 2 (Inspektor Research Systems BV; Amsterdam, Netherlands). To locate the specimens in the same angle and same camera position, the QLF-D was affixed to the QLF-D *in vitro* stand. The video-repositioning software of the QLF-D system was used to ensure that images are automatically captured when the correlation is higher than 0.9. Before the measurements, the specimens were dried for 5 s with an air syringe. In addition, to ensure that the specimens were dried equally, measurements of each enamel specimen were performed after 15 minutes. All images were captured in a dark room with no ambient light. For each specimen, the relative fluorescence loss (ΔF %) (the difference in the intensity of the green fluorescence of the sound enamel and of the demineralized lesion) was calculated at pretreatment phase (baseline), 1 week after the treatment, and 4 weeks after the treatment. During the analysis, a contour was drawn around the artificial caries lesion area using the White Spot Wizard. The contour of the area was marked on the original, sound enamel. The difference between the sound enamel (the reference area) and the lesion area was calculated, and quantitative results were obtained for the following parameters: mean fluorescence loss over the lesion (ΔF %) and area of the lesion (pixel^2^).

### Measurements with LF pen

The LF pen (DIAGNOdent 2190; KaVo; Biberach, Germany) measurements were performed using a cylindrical, sapphire-fiber tip, which is specifically designed for smooth surfaces, according to the manufacturer’s instructions. The examiner had been experienced in using and handling the device before the measurements were done. The device was calibrated against a ceramic standard, and it was recalibrated after testing 10 specimens. After calibration, each block was dried with a paper tissue and air-dried for 5 s. Each block was analyzed 3 times and the mean values were calculated. The fluorescence values for each specimen were measured in 4 steps: at baseline, after demineralization, 1 week after remineralization, and 4 weeks after remineralization.

### SMH measurements

The microhardness of the 30 specimens was measured for 15 s using a Vickers microhardness tester (Shimadzu Micro Hardness Tester HMV-2; Shimadzu Corporation; Kyoto, Japan) with a load of 200 g. All readings were performed by the same examiner using the same calibrated machine. For each enamel specimen, the average value of three indentation scores was used to represent the specimen’s hardness value. The microhardness values for each specimen were measured in 4 steps: at baseline, after demineralization, 1 week after remineralization, and 4 weeks after remineralization.

### EDS analysis

Following the QLF-D, LF pen, and SMH measurements, the samples were left to air dry at room temperature for 24 h. Then, before EDS examination, the teeth were sputter-coated with gold-palladium (EMITECH K550X Sputter Coater; Emitech Ltd.; Ashford, UK) and the samples were placed in the vacuum chamber. EDS examination was performed using a scanning electron microscope (Quanta-FEG 250; FEI Co.; OR, USA) equipped with an EDS detector (with spot size at 3.0 and voltage at 10 kV) to assess mineral content. For each enamel slab, the mean calcium (Ca), phosphate (P), and fluoride (F) content and the Ca/P ratio were calculated.

### Statistical analysis

The SMH, QLF-D, EDS, and LF pen scores were analyzed using the IBM SPSS Statistics version 20.0 statistical package (SPSS; Chicago, IL, USA). The normality of the data was confirmed using the Shapiro-Wilk test. The repeated measures ANOVA test was used to compare the mean values at the various time points, and the significance level was set at 0.05 (α=0.05).

## RESULTS

Table 1 presents the mean QLF-D values for lesion area and fluorescence loss. There were significant differences among three phases, and the fluorescence loss was significantly reduced after both one- and four-week treatment periods. The mean lesion area values in the remineralization phases were significantly lower than the baseline values (p<0.05). However, the difference between the two remineralization times was not significant (p>0.05*)*.

**Table 1:** Summary statistics for fluorescence loss and lesion area for each time point. Entries in each cell are mean and standard deviation (n=30)

ΔF (%)			Area (pixel^2^)		
Baseline	1-week	4-weeks	Baseline	1-week	4-weeks
-44.20^a^	-32.24^b^	-26.35^c^	2173.17^a^	1925.90^b^	1844.17^b^
(5.7)	(5.7)	(9.7)	-254.72	-284.3	-272.84

ΔF: Change in fluorescence

Different lowercase letters indicate significant differences between different time points (p<0.05)

Table 2 presents the mean SMH and fluorescence values for the LF pen and the standard deviations of the enamel in each measurement phase. With regard to the SMH analysis, differences among the four phases were statistically significant (p<0.05). The SMH values of the demineralized enamel specimens differed significantly compared with baseline values (p<0.05) and increased significantly after both the one- and the four-week treatment periods (p<0.05). The LF pen measurements showed significant differences between measurements made at baseline, after demineralization, and after one week of remineralization, with the highest values being obtained for the demineralization phase (p<0.05). However, the difference between the one-week and the four-week time points was not significant.

**Table 2:** Surface microhardness values for microhardness test and fluorescence values for LF pen in different time points (mean ± SD) (n=30)

	Baseline	Demineralization	1-week	4-weeks
SMH	267.80 ± 17.29^a^	60.96 ± 6.80^b^	92.60 ± 19.81^c^	113.78 ± 19.57^d^
LF pen	4.13 ± 0.40^a^	21.17 ± 2.21^b^	18.21 ± 3.38^c^	17.82 ± 2.72^c^

Significant differences are indicated within same row by different superscript letters (p<0.05)

Table 3 shows the results of the EDS analysis. After the one- and four-week treatment periods, Ca and P concentrations and the Ca/P ratio were higher compared to those of the demineralized surfaces (p<0.05). While there was no significant difference between the demineralization and one-week treatment phases, after the four-week treatment period the F content was significantly higher compared with that in the demineralized areas (p<0.05).

**Table 3:** Weight percentages of calcium, phosphorus, fluoride content and the Ca/P ratio of enamel according to the different time points (mean ± SD) (n=30)

	Baseline	Demineralization	1-week	4-week
Ca	39.43 ± 2.03^a^	32.18 ± 2.41^b^	35.99 ± 3.42^c^	36.89 ± 3.52^d^
P	17.95 ± 0.52^a^	16.14 ± 0.91^b^	17.09 ± 0.82^c^	17.29 ± 1.03^d^
F	0.07 ± 0.09^a^	0.00 ± 0.00^b^	0.02 ± 0.04^a,b^	0.05 ± 0.08^a^
Ca/P	2.20 ± 0.08^a^	1.99 ± 08^b^	2.10 ± 0.12^c^	2.13 ± 0.11^c^

Different lowercase letters indicate significant differences within same row (p<0.05)

## DISCUSSION

Increasing demand for noninvasive treatment of WSLs has led dental researchers to focus on developing materials that have greater remineralization ability than the conventional agents. Among these, materials containing CPP-ACP have become popular for remineralizing incipient caries lesions^4^ ^,^ ^7^ ^,^ ^10^ ^,^ ^21^ ^,^ ^27^. Although numerous studies related to CPP-ACP paste have demonstrated the remineralizing potential of the agent, to our knowledge, there has been limited study of the efficacy of CPP-ACP containing fluoride varnish for remineralizing WSLs. Therefore, in the present study, an *in vitro* model was used to compare the remineralizing efficacy of CPP-ACP containing fluoride varnish on artificial caries lesions. The effects of the material were assessed using SMH, QLF-D, EDS, and LF pen.

The mineral loss or gain in enamel caused by demineralization or remineralization can be measured by evaluating changes in the SMH of the enamel. SMH measurements provide a relatively simple, nondestructive, and rapid method of assessing demineralization and remineralization^28^. According to the results of this study, significant differences were found between the baseline, demineralization, and post-treatment SMH measurements, and the varnish treatment significantly increased the SMH of the demineralized enamel. Previously, several studies have investigated the synergistic effect of CPP-ACP and fluoride on caries lesions, and one study assessed the ion release of MI Varnish; the results obtained were consistent with those of previous studies^4^ ^,^ ^5^ ^,^ ^7^ ^,^ ^10^ ^,^ ^21^ ^,^ ^27^. Cochrane, et al.^5^ (2014) suggested that the ion-release profile of MI Varnish was the most promising, as it had greater fluoride and calcium ion release compared with other varnishes containing calcium fluoride, tricalcium phosphate, amorphous calcium phosphate, and fluoride^5^. Researchers explained the rapid ion release from MI Varnish to the high water solubility of the CPP-ACP complexes: It is thought that the great remineralization ability of MI Varnish may be caused by this rapid release of ions from the material and by the bioavailable nature of the CPP-ACP contained in the varnish.

Because the conventional methods of monitoring the mineral loss or gain occurring as a result of demineralization and remineralization are not possible to be used clinically, quantitative methods that are clinically applicable have been developed to detect and monitor caries in the mineral content. One such method, QLF, is a visible light system that can quantitatively detect the degree of demineralization and afterwards monitor its progression or regression nondestructively^13^ ^,^ ^28^. Previous studies have tested the QLF method in *in vitro* experiments with transverse microradiography (TMR), optical coherence tomography (OCT), scanning electron microscopy/energy dispersive X-ray spectroscopy (SEM/EDS), SMH tests, and digital photography^2^ ^,^ ^3^ ^,^ ^14^ ^,^ ^17^. These studies have reported that QLF can be used to monitor the progression or regression of enamel demineralization. The QLF-D Biluminator^™^ 2 used in this study is an upgraded version of the first product that has a modified filter set (D007; Inspektor Research Systems BV; Amsterdam, Netherlands) and has other upgrades to enhance the characteristics of the QLF. In QLF-D analysis, significant differences were found between demineralization and post-treatment QLF-D measurements. In addition, with regard to fluorescence gain, there was a significant difference between the one- and four-week treatments, and fluorescence gain was increased gradually. These findings suggested that a single application of fluoride varnish containing CPP-ACP causes a significant increase in fluorescence and a reduction of the lesion area.

LF is a method of detecting caries and monitoring the mineral loss or gain of tooth structure. The DIAGNOdent LF pen is capable of capturing, analyzing, and quantifying the fluorescence changes in the tooth structure^21^. In the present study, the LF pen fluorescence values showed significant differences among the three phases (baseline, after demineralization, and after one week of remineralization), but there was no significant difference between the first and fourth weeks. These findings suggested that MI Varnish is an effective agent for remineralizing of WSLs. However, the inconsistent findings between SMH and QLF analyses with LF pen for 1- and 4-week remineralization changes may be explained by the fact that the LF pen device is not capable of detecting small changes in mineral content of WSLs. Some studies evaluating the performance of LF pen in monitoring remineralization have obtained conflicting results regarding detecting and monitoring of WSLs, and little evidence is available about its efficacy in monitoring remineralization in incipient caries^6^ ^,^ ^11^ ^,^ ^15^ ^,^ ^16^ ^,^ ^23^. In the current study, LF pen was found to be an appropriate device for detecting demineralization and larger remineralization changes; however, it was not efficient in detecting small remineralization changes under *in vitro* conditions.

The remineralization process is dependent on the mineral changes in the structure of dental hard tissues. The levels of Ca, P, and fluoride elements in enamel represent an indication of the rate of demineralization or remineralization, and quantifying these levels can denote lesion progress^9^ ^,^ ^11^ ^,^ ^17^. In this study, EDS was used to evaluate the changes generated by the remineralization agent on the demineralized enamel. EDS is a powerful instrument that performs quantitative elemental analyses by measuring the characteristics of re-emitted X-rays, and using this device can help to compare the effects of various materials on demineralized enamel samples^17^. In the present study, Ca and P content and the Ca/P ratio were significantly higher when remineralization values were compared to demineralized areas after one- and four-week treatments. This result indicates the remineralization potential of the agent used in the study.

In the present study, the remineralizing efficacy of MI Varnish was evaluated by analyzing enamel using SMH, QLF-D, EDS, and LF pen. The results clearly demonstrated that MI Varnish is an effective agent and useful for remineralizing of WSLs. Although the complex oral environment could not be entirely mimicked in the present study, the results provided useful information about the effectiveness of the tested agent for remineralizing of enamel. Future *in vivo,* comparative studies, and *in vitro*/*in vivo* studies should be designed to assess the efficacy of the material over long periods.

## CONCLUSIONS

Under the limitations of the present study, the following conclusions can be made:

1. CPP-ACP containing fluoride varnish provides remineralization of incipient carious lesions after a single application and seems suitable for clinical use.
2. An upgraded QLF-D device seems helpful in *in vitro* studies and provides quantitative, consistent results.
3. The LF pen system is an appropriate device for detecting demineralization processes and larger remineralization changes; however, it has limited ability to detect small remineralization changes under *in vitro* conditions.
